# Norms and the Flexibility of Moral Action

**DOI:** 10.1017/pen.2018.13

**Published:** 2018-09-07

**Authors:** Oriel FeldmanHall, Jae-Young Son, Joseph Heffner

**Affiliations:** Department of Cognitive, Linguistic, and Psychological Sciences, Brown University, Providence, RI, USA

**Keywords:** moral, norms, reciprocity, emotions, learning

## Abstract

A complex web of social and moral norms governs many everyday human behaviors, acting as the glue for social harmony. The existence of moral norms helps elucidate the psychological motivations underlying a wide variety of seemingly puzzling behavior, including why humans help or trust total strangers. In this review, we examine four widespread moral norms: Fairness, altruism, trust, and cooperation, and consider how a single social instrument—reciprocity—underpins compliance to these norms. Using a game theoretic framework, we examine how both context and emotions moderate moral standards, and by extension, moral behavior. We additionally discuss how a mechanism of reciprocity facilitates the adherence to, and enforcement of, these moral norms through a core network of brain regions involved in processing reward. In contrast, violating this set of moral norms elicits neural activation in regions involved in resolving decision conflict and exerting cognitive control. Finally, we review how a reinforcement mechanism likely governs learning about morally normative behavior. Together, this review aims to explain how moral norms are deployed in ways that facilitate flexible moral choices.

Consider the various social norms that dictate how you behave in your daily life: You refrain from having conversations in a theatre, you dress conservatively in a place of worship, you tip your waiter after good service, and you keep a secret when a friend tells you something confidential. Humans share a set of social and moral beliefs that govern how we behave, from mundane chitchat during a movie to the most consequential behaviors that dictate whether we harm or help others. Norms help create social cohesiveness and an understanding of shared expectations that support and shape identities at both a societal and individual level. For this reason, norms are critically important for determining whether social communities function well and efficiently.

Humans rely on a set of complex, evolved, and learned norms to encourage community members to adopt certain perspectives that can guide and promote prosocial interactions (Cialdini, 2003; Cialdini, Reno, & Kallgren, 1990; Goldstein, Cialdini, & Griskevicius, 2008; Nichols, 2004; Sherif, 1936). These moral norms are so important for social functioning that there appears to be a sort of universal moral grammar, through which certain moral norms are sacredly held. This has been demonstrated in multiple research fields (Bicchieri, 2006), including affective neuroscience (Chang & Smith, 2015), cognitive development (Kohlberg & Hersh, 1977; Van de Vondervoort & Hamlin, 2018), cross-cultural studies (Hauser, 2006; Mikhail, 2007), and work on nonhuman primates (de Waal, 2009). The growing literature suggests that this seemingly elaborate system of natural jurisprudence is relatively stable over time and extends across social groups (Mikhail, 2008; Sripada, 2008).

Here we discuss the importance of social and moral norms, what types of values they convey, and how their existence can alter behavior. We begin by defining social and moral norms: What are they, how do they develop, and how are they sustained? We then go on to discuss the set of moral norms we consider to be foundational for harmonious and successful social living. We propose that a single mechanism—reciprocity—underpins the adherence to, and enforcement of, most moral behaviors. Using a game theoretic perspective, we illustrate how these norms act as a driving force behind flexible moral behavior (Melnikoff & Bailey, 2018), whereby different classes of behavioral patterns can arise depending on which norm is activated (Ajzen, 1991). We review neural evidence that people find it intrinsically rewarding to comply with moral norms, before examining how emotions can enhance reciprocal behaviors and the adherence to moral norms. Finally, we discuss how moral norms are likely learned and sustained through reward and punishment contingencies based on expectations of reciprocity.

## 1. What are social and moral norms?

Social norms are ubiquitous and endemic to social life. They provide a standard for behavior based on mutual and widely shared psychological attitudes, expectations, and beliefs about how members of society ought to behave (House, 2018). At the broadest level, these norms help to promote harmonious living, in which the concerns of others are taken into account (Ullmann-Margalit, 1978). They prescribe mores (e.g., wear black to a funeral) and sometimes even consequential rules (e.g., while in America, drive on the right side of the road) about what people should and should not do in various social situations (Turiel, 1983). Since deviations from social norms often elicit informal (or even formal) social sanctions, they are a useful explanatory tool for describing many of our everyday social behaviors.

Moral norms can be considered a subset of social norms in that they explicitly govern behaviors that have positive or negative outcomes for both the self and others. For example, social norms, such as “do not chew gum at the table,” typically appeal to a wide set of behaviors without necessitating harm be prevented (Turiel, 1983). In contrast, moral norms, such as “behave fairly,” dictate that individuals navigate through the world without harming others (Schein & Gray, 2017). In some cases, moral norms act in opposition to ingrained desires (e.g., biological urges), which are generated to promote survival (Darwin, 1859; Dawkins, 1989). Enhancing self-benefit—for example, increasing one’s wealth, power and prestige—is one avenue by which biological urges can be expressed. If increasing one’s wealth leads an individual to deviate from morally normative patterns, negative consequences for others may ensue (harm is applied, money stolen, and so forth). The existence of moral norms, which aim to promote the well-being of others and the community at large, can help attenuate these negative consequences by tempering (either through suppression or regulation of) these self-enhancing desires.

Accordingly, if a core component of morality is that humans share a set of codes and beliefs that dampen selfish inclinations, it is important to examine what those moral strictures might be. We make the case that there are four fundamental moral norms—fairness, altruism, trust, and cooperation—that play a prominent role in shaping many everyday social interactions. While there are other possible candidate norms that could be included (e.g., norms of respect, justice, harm, and so forth), these four norms are sufficiently general enough to be applicable to a wide array of moral behavior (e.g., trusting that an individual will not be harmed by others), while also having enough specificity to capture unique behavioral patterns across them. Here we argue that these norms of fairness, altruism, trust, and cooperation are all subserved by, and rooted in, a single mechanism—reciprocity—that enables people to make flexible moral decisions across a range of social contexts.

### 1.1. Reciprocity as a mechanism

Reciprocity has traditionally been operationalized either as individual beliefs about the structure of the world, or as a culturally mandated standard of behavior. In regards to the first, reciprocity is often construed within a framework of a “just world” (Lerner, 1980), whereby people believe in a system of social exchanges that reach a fair equilibrium over time (Gouldner, 1960). Such a belief in universal justice implies that destructive individuals who violate expectations of reciprocity will eventually face consequences for disturbing the equilibrium. On the other hand, from a culturally mandated normative standpoint, reciprocity is widely perceived as a moral “ought” (Eisenberger, Lynch, Aselage, & Rohdieck, 2004; Gouldner, 1960; Tsui & Wang, 2002). This framework argues that reciprocity operates either by responding to negative actions with negative treatment, or by responding to positive actions with positive treatment. One particularly potent example of negative reciprocity is when punishment is levied on those who do not comply with moral norms (Fehr & Fischbacher, 2004; Gintis, 2000).

However, instead of viewing reciprocity as a norm in and of itself, it may be more appropriate to refashion the concept of reciprocity as a mechanism that motivates adherence to a suite of moral norms (Cropanzano & Mitchell, 2005; Leimgruber, 2018). From this framework, reciprocity can powerfully and flexibly drive different behaviors, from rewarding those who help, to punishing those who harm (Dufwenberg & Kirchsteiger, 2004; Gintis, Henrich, Bowles, Boyd, & Fehr, 2008; Gouldner, 1960; Nowak, 2006; Rabin, 1993; Rand & Nowak, 2013). Below, we discuss the roles that contextual factors, emotional experiences, and learning play in influencing how reciprocity supports flexible moral action.

## 2. The norms that govern moral behavior

In the following section, we use a game theoretic approach to examine fairness, altruism, trust, and cooperation. This is done for two reasons. First, behavioral economic games allow researchers to observe how individuals anticipate, infer, and act on what others do (Von Neumann & Morgenstern, 1945). As each game has a series of discrete rule sets, researchers can control and manipulate the structure and context of any game (Camerer, 2003). To the extent that people’s decisions are exquisitely sensitive to the intricacies and contextual minutia of the game environments, researchers can observe how different norms and expectations alter social and moral behavior by modifying the games’ rule sets. Second, the strategic interactions and behaviors that fall out of economic games are mathematically expressed on a universal scale: We know with precision how much money an individual is willing to altruistically offer another, how much punishment is conferred upon a perpetrator in the wake of a fairness violation, and how much people care to trust or cooperate with an unfamiliar partner. Together, these two dimensions of economic games provide a powerful testbed for understanding moral norms and their influence on behavior.

### 2.1. Fairness

It is difficult to imagine how groups of individuals would manage to divide resources in a harmonious way without appealing to a shared standard of fairness (Charness & Rabin, 2002; Fehr & Schmidt, 1999). Although resources can be divided meritoriously (e.g., according to an individual’s effort or contribution), the overarching norm of fairness mandates that, all things considered, resources ought to be divided equitably among community members. Evidence for this fairness norm is abundant. Strangers routinely split resources equitably in the absence of social sanctions (Fehr & Fischbacher, 2003; Roth, Prasnikar, Okuno-Fujiwara, & Zamir, 1991; Zelmer, 2003), notions of fairness are universally appealed to across cultures (Henrich et al., 2005, 2010), human infants are sensitive to (and expect) the equitable distribution of resources (Sommerville & Enright, 2018), and even nonhuman animals (e.g., primates, dogs, and birds) are attentive to unequal outcomes between members of their own species (Brosnan & de Waal, 2014).

How humans resolve fairness transgressions has been a central question in behavioral economics for decades. Economists have traditionally used the Ultimatum Game (Güth, Schmittberger, & Schwarze, 1982) to demonstrate that after experiencing a fairness violation (Bicchieri & Chavez, 2010), people are willing to forgo even large sums of money to punish norm violators (Camerer, 2003). In the Ultimatum Game, two players partake in an economic exchange. One player acts as the Proposer and makes an offer to the other participant, the Responder. The Responder can then either accept or reject the offer. If accepted, the money is split as proposed. If rejected, then neither player receives any money, which effectively punishes the Proposer for offering an unfair split. The most rational decision for the Responder is to accept any offer from the Proposer no matter how small, since some money is better than no money. However, people routinely go against monetary self-interest to reject unfair offers (Fehr & Fischbacher, 2004; Fehr & Gächter, 2002; Henrich et al., 2006; Herrmann, Thöni, & Gächter, 2008; Jordan, Hoffman, Bloom, & Rand, 2016; Sanfey, Rilling, Aronson, Nystrom, & Cohen, 2003), indicating a strong preference for upholding fairness norms.

Despite this preference, individuals frequently split resources unequally to benefit themselves, revealing a dueling desire to act in one’s own self-interest (Camerer & Thaler, 1995; Kahneman, Knetsch, & Thaler, 1986). This class of unfairness is termed advantageous inequality (when one receives more than another), which stands in contrast to disadvantageous inequality (when one receives less than another). Such unequal distributions are not ideal for lasting partnerships, since receiving less than another signals a disadvantageous relationship that should potentially be terminated, while receiving more reward might risk undermining future goals because one’s partner could feel exploited (Nishi, Shirado, Rand, & Christakis, 2015). Accordingly, although self-interest may initially lead an individual to prefer advantageous inequality, minimizing both types of unfairness helps individuals and societies stabilize long-term ventures (Piketty, 2017; Tavoni, Dannenberg, Kallis, & Löschel, 2011). The mutual expectation of fair treatment therefore leads individuals on both sides of the dyad to prefer equal payoffs.

As a consequence, concerns about maintaining fairness create incentives for individuals to punish those who violate fairness norms, even if the transgression does not affect one’s own welfare. Indeed, the desire to punish is so strong that even third-party members—who have no clear vested interest in the equal distribution of resources between others—are still willing to incur a cost to ensure that those peddling unfair allocations are punished (Cronk, Chagnon, & Irons, 2000; Fehr & Fischbacher, 2004). This may be due in part to the notion that failing to punish can itself be punishable (Kandori, 1992).

Decisions to punish unfairness—whether as a victim or third-party member—ultimately demonstrate negative reciprocity, whereby the amount of punishment approximately equals the harm caused (Carlsmith, Darley, & Robinson, 2002; Fehr & Gächter, 2000a, 2000b). As a mechanism for enforcing equality, negative reciprocity encourages individuals to offer fair distributions (Azar, Lahav, & Voslinsky, 2015). Furthermore, people who engage in negative reciprocity can procure positive reputational benefits (Gintis, Smith, & Bowles, 2001). For example, individuals who punish are trusted more, and in return, behave in a more trustworthy manner (Jordan et al., 2016). In contrast, the threat of public exposure of unfair behavior (and thus the possibility of accruing a negative reputation), leads individuals to make more fair offers (Bolton & Zwick, 1995; Straub & Murnighan, 1995). These findings illustrate that negative reciprocity, through punishment, helps enforce and maintain norms of fairness, and by extension the overall well-being of social communities (Gürerk, Irlenbusch, & Rockenbach, 2006; Herrmann et al., 2008).

### 2.2. Altruism

Some accounts of natural selection argue that survival requires self-benefit be prioritized at all costs. At first blush, acts of altruism—choosing to help another at a cost to the self (de Waal, 2008)—seem to significantly reduce one’s evolutionary fitness (Darwin, 1859). However, an influential concept known as “kin selection” posits that altruistic individuals’ genes propagate when prosocial behaviors are performed, which aids in the survival of genetically related individuals (Trivers, 1971; Wilson, 2000). Accumulating evidence now demonstrates that altruistic behavior is not confined to kin selection strategies, and many species expend valuable resources to help unrelated others (FeldmanHall, Mobbs, et al., 2012; Pitman et al., 2017; Preston & de Waal, 2002; Quervel-Chaumette, Dale, Marshall-Pescini, & Range, 2015; Warneken, Hare, Melis, Hanus, & Tomasello, 2007). Given these findings, it has been subsequently argued that altruism may have evolved for the good of the social community and not just individual genes (Nowak, Tarnita, & Wilson, 2010).

Applying a slight modification to the Ultimatum Game described above elegantly illustrates this point. If the option to reject the offer is removed, the social exchange becomes a Dictator Game (Camerer, 2003) where the Receiver must accept any offer no matter how small. Although the rational decision is for a Dictator (analogous to the Proposer in the Ultimatum Game) to offer the smallest amount of money (since the split is always realized as-is), Dictators routinely go against such monetary self-interest and offer around 28% of their initial endowment (Engel, 2011). Critically, these acts of generosity observed in the lab reflect real-world concerns for altruism (Benz & Meier, 2008; Kosfeld, Heinrichs, Zak, Fischbacher, & Fehr, 2005; Moll et al., 2006). In America, approximately 60% of households give to charity each year (at a rate of about 4% of a household’s income), which totals to more than $250 billion a year (Meer, Miller, & Wulfsberg, 2017).

On the surface, charitable giving does not seem to be a self-beneficial act. Money is given to unknown others, oftentimes in distant countries where there is little chance of meeting those who received the donation. In these cases, it is unlikely that one’s altruistic behavior will be directly reciprocated by that specific individual. However, when viewed through the lens of an indirect reciprocity mechanism, the existence of altruistic behaviors has important implications for how we expect humans to behave and be treated in a community (Simpson & Willer, 2008). One example is that individuals hold expectations that people will be behave in generous ways (Brañas-Garza, Rodríguez-Lara, & Sánchez, 2017), and violating these expectations may result in punishment by third-parties (Fehr & Fischbacher, 2004). Thus, acting selflessly by donating to others provides an advantage to the altruist in that there will be some kind of indirect, downstream benefit (or avoidance of admonishments) from the community at large.

Compellingly, human social groups that act altruistically appear to fare better than those who do not (Ostrom, 2014). Take welfare states for example: Even though many Western societies are large and complex, members are intimately dependent on one another, as there are social expectations that people who are more fortunate will help those who are less fortunate (Wilensky, 1974). This norm of altruism ranges from long-term governmental edicts to fleeting one-on-one relationships (Barr, 2012). If an individual is drowning in a lake or falls off a subway platform, people nearby will even risk their lives to help the distressed individual (Marsh et al., 2014). These acts of altruism are typically performed without the belief that the beneficiary will directly return the favor. Rather, the expectation (even if implicitly held) is that someone else will display a similarly altruistic act if the altruist were later in a situation and needed help (Nowak & Sigmund, 1998, 2005). Indeed, removing the ability to directly reciprocate a generous act can motivate individuals to “pay it forward” by helping another in need (Gray, Ward, & Norton, 2014; Hackel & Zaki, 2018).

Such costly indirect altruism is believed to be a key factor in the evolution of human cooperation (Nowak, 2006), and simulations of Dictator Game behavior reveal that indirect and direct generosity is driven by the anticipation of such uncertain future relationships (Delton, Krasnow, Cosmides, & Tooby, 2011; Zisis, Di Guida, Han, Kirchsteiger, & Lenaerts, 2015). This is in part believed to be causally influenced by feelings of moral obligation, social responsibility (Schwartz, 1977), and the knowledge that others are behaving in generous ways (Bartke, Friedl, Gelhaar, & Reh, 2017). For example, activating norms of altruism induces greater helping, and fluctuations in the environment (e.g., the level of a target’s expressed distress or number of individuals who can readily help) can either amplify or attenuate altruistic decisions (Cameron & Payne, 2011; Darley & Batson, 1973; FeldmanHall, Dalgleish, Evans, & Mobbs, 2015; Gottlieb & Carver, 1980; Preston, 2013; Preston & de Waal, 2002). Other motivations, such as the desire for social prestige and reputation (Olson, 1965), or avoiding social ostracism (Becker, 1974), are also known to influence altruism and can be considered positive or negative reciprocity, respectively.

### 2.3. Trust

Trust spans a multitude of situations, cultures, and disciplines, as it is an integral feature of relationships between spouses, friends, teachers and students, and governments and civilians (Cottrell, Neuberg, & Li, 2007). Trust facilitates positive social interactions and has been suggested to be one of the foundations of an efficient economy; there is a strong correlation between economic growth and the percentage of citizens who generally trust others (Knack & Keefer, 1997). This is unsurprising given that a significant aspect of any economic transaction is the ability to trust and cooperate with nonrelated others (Arrow, 1974). At the dyadic level, deciding to trust—lending money to a friend or sharing personal information with an acquaintance—allows for the formation of partnerships that can produce mutual advantages to maximize an individual’s social fitness (Trivers, 1971) and overall societal well-being (Fehr & Camerer, 2007). However, decisions to trust are inherently risky because of the unpredictability and uncertainty of partners’ responses during social exchanges (Vives Moya & FeldmanHall, 2018). For example, an untrustworthy individual may fail to repay a loan, or gossip about another’s personal information. Without trust, however, neither markets nor social relations could thrive, as there would be an unwillingness to risk something of value in exchange for a later reward.

As with fairness and altruism, trust can be measured using a simple economic game (Berg, Dickhaut, & McCabe, 1995). A typical Trust Game involves a one-shot social interaction between two players, an Investor and a Trustee. The first player, the Investor, is initially faced with a decision to keep an endowment of money (e.g., $10) or share part of it with the Trustee. If shared, the investment is multiplied (often by a factor of four), and the Trustee faces the difficult decision to repay the trust by sending back up to half of the increased sum, or to violate that trust by keeping all the money, leaving the Investor with nothing. The social dilemma for the Investor is clear: Though it is more profitable to trust if it will be reciprocated, doing so leaves the Investor susceptible to the risk of a breach in trust, and ultimately, the loss of money. This game can be adapted for repeated play, such that social sanctions, communication between players, reputation, and relationships can all be manipulated.

In traditional formulations, the Investor normally trusts approximately 50% of their endowment to a Trustee, and the Trustee typically returns 50% of the expanded pie (Camerer, 2003). In one-shot games where there is no opportunity for social sanctions or reputation building through repeated play, it is rather surprising that Trustees return so much of the money, especially since many economists would argue that a rational, self-interested person should return nothing. That Trustees do not exhibit this behavioral pattern—even in situations where individuals are playing together only once and doing so anonymously—suggests the existence of (and adherence to) a moral norm of reciprocal trust (Cox, 2004; Dunning, Anderson, Schlösser, Ehlebracht, & Fetchenhauer, 2014; McCabe, Rigdon, & Smith, 2003). Moreover, if Trustees were only motivated by altruistic generosity, then their typical return should map onto the 28% given by Dictators in the Dictator Game (Engel, 2011). Thus, the expectation of direct reciprocity, the critical component of any Trust Game, appears to exist on both sides of the dyad: an individual invests her money because she believes that it will be reciprocated (Ma, Meng, & Shen, 2015), and partners reciprocate the increased monetary sum because there is a strong expectation of reciprocity (Baumgartner, Fischbacher, Feierabend, Lutz, & Fehr, 2009; Chang, Smith, Dufwenberg, & Sanfey, 2011; Fareri, Chang, & Delgado, 2012, 2015).

Of course, the degree to which individuals value norms of trust can vary. Even when the parameters of a task are held constant, there are some individuals who resolutely adhere to reciprocal trust norms and others who deviate from this norm (Baumgartner et al., 2009; Cesarini et al., 2008). There are other cases in which individuals might doggedly reciprocate trust in one situation, but swiftly forgo reciprocal behavior when the situation changes (Melnikoff & Bailey, 2018). For example, if a Trustee knows an Investor made a highly risky decision to trust, the Trustee will reciprocate with more money, illustrating the exquisite sensitivity people have to normative signals (Van Den Bos, van Dijk, Westenberg, Rombouts, & Crone, 2009). Evidence that individuals behave in accordance with, or deviate from, a moral norm depending on the context, suggests that adhering to a moral code of trust can be quite malleable.

### 2.4. Cooperation

Norms can also powerfully influence cooperative behavior (Ostrom, 2014). Behavior in one-shot cooperation problems such as the Prisoner’s Dilemma or Public Goods Game reveals that people typically cooperate, despite understanding that it is in one’s best self-interest to maximize reward by defecting (Andreoni & Miller, 1993; Blake, Rand, Tingley, & Warneken, 2015; Harrington, 1995; Sally, 1995). From a purely economic perspective, this is a puzzling behavior, as it suggests that the social norm of cooperation is more motivating than maximizing favorable outcomes for the self. Even group size (i.e., playing with four individuals as opposed to 100) does not substantially change the rate at which individuals cooperate (Isaac, Walker, & Williams, 1994), indicating that there is a desire to maintain cooperation even in large, anonymous, and complex settings.

Prominent theories suggest that cooperation exists because of a reciprocal tit-for-tat pattern of behavior (Fehr & Fischbacher, 2004; Fehr & Gächter, 2000a, 2000b; Hamilton & Axelrod, 1981). Once there is an initial signal to cooperate, others will cooperate in return. This notion of conditional cooperation is supported by strong empirical evidence: When communication of intentions is allowed between partners, high levels of cooperation follow suit (Bohnet & Frey, 1999; Messick & Brewer, 1983; Ostrom & Walker, 2003; Sally, 1995). In contrast, cooperation languishes when external rules and sanctions are directly and explicitly imposed, compared with systems that allow internal norms to spontaneously develop (Yamagishi, 1988). These cooperative patterns can be manipulated by expectations of either direct or indirect reciprocity. Repeated play, for instance, typically garners greater rates of cooperative behavior (Fudenberg, Rand, & Dreber, 2012; Nowak, Sasaki, Taylor, & Fudenberg, 2004; Rand & Nowak, 2013). In these cases, individuals form a belief that their fellow partners will cooperate if they cooperate, a form of direct reciprocity. Cooperation can also arise out of indirect reciprocal actions, such as when an individual cooperates knowing that other individuals will be privy to this information (Gächter & Fehr, 1999; Mao, Dworkin, Suri, & Watts, 2017). Such a system allows individuals to enhance their reputation by cooperating more, thereby procuring the downstream benefits that are associated with positive social standing (Pfeiffer, Tran, Krumme, & Rand, 2012).

As with other norms, patterns of cooperation can vary depending on the setting (Hilbe, Chatterjee, & Nowak, 2018; Ostrom, 2014). Contextual factors—such as whether others around you are cooperating (Fowler & Christakis, 2010; Mao et al., 2017), whether the norm of cooperation has been primed (Capraro, Smyth, Mylona, & Niblo, 2014; Peysakhovich & Rand, 2015), whether resources are abundant (Van Vugt & Samuelson, 1999), whether the size of temptation to freeride is small (Van Lange, 1992), or whether an individual is a member of a collectivist culture where there are strong norms of reciprocity amongst in-group members (Hofstede, 1980; Leung, 1997)—all positively contribute to an individual ultimately cooperating. Importantly, many of these contextual factors can also shape perceptions of reciprocity. For example, when social cues are available (e.g., discussing strategies with a partner before starting the game), the likelihood of reciprocity rises by as much as 40% (Sally, 1995). In contrast, when there is uncertainty within the environment (e.g., ambiguity around the size of the resource being split, or how many members are using the resource), it reduces an individual’s willingness to cooperate (Budescu, Rapoport, & Suleiman, 1990, 1992; Budescu, Suleiman, & Rapoport, 1995).

## 3. Norm compliance is rewarding

From a decision-making perspective, an individual who chooses to comply with moral norms demonstrates that the value of norm compliance is greater than the value of selfishly maximizing one’s self-benefit. In this section, we evaluate evidence from the neuroimaging literature that demonstrates how norm compliance and reciprocal behaviors systematically engage the brain’s reward network.

The clearest neural evidence that people value reciprocity comes from studies on trust, cooperation, and fairness. In the domain of trust, neuroimaging experiments utilizing the Trust Game illustrate that the caudate, a region critical for indexing reward, computes information about the intention to reciprocate trusting acts (King-Casas et al., 2005), and that other regions within the reward network—most notably, the ventral tegmental area (VTA) and ventral striatum—subserve reciprocal exchanges of trust between two players (Krueger et al., 2007; Phan, Sripada, Angstadt, & McCabe, 2010). A recent meta-analysis further reveals that these value signals are likely to be linked to aspects of reciprocity rather than to trust itself. When deciding to trust in repeated games (where direct reciprocity has the opportunity to manifest), there is a high likelihood that ventral striatum is recruited, but not in one-shot games, where direct reciprocity is impossible (Bellucci, Chernyak Sergey, Goodyear, Eickhoff Simon, & Krueger, 2016). Indeed, reciprocation of trust can be experimentally increased by stimulating the right orbitofrontal cortex suggesting that reward regions contribute critically to reciprocal actions (Wang, Li, Yin, Li, & Wei, 2016). The value associated with reciprocal trust also appears to be conditional on social distance. Individuals trust close friends with more money than strangers (even when friends and strangers reciprocate at the same rate), which is associated with greater ventral striatum activity (Fareri et al., 2015). Thus, not only does reciprocity appear to depend on immediate observations (i.e., did my partner just behave in a way that reciprocated my trust?), but it also seems linked to previously learned expectations (i.e., is my partner generally someone who would reciprocate my trust?).

Studies of reciprocal cooperation demonstrate a similar engagement of reward-processing regions. An early experiment using the Prisoner’s Dilemma observed that mutual cooperation was reported as highly satisfying, and these cooperative decisions were associated with enhanced blood-oxygen-level-dependent activity within in the nucleus accumbens, caudate, and orbitofrontal cortex (Rilling et al., 2002). Subsequent work contrasting neural responses in cooperative and competitive variants of a coordination game found that mutual cooperation recruits orbitofrontal cortex (Decety, Jackson, Sommerville, Chaminade, & Meltzoff, 2004) even when coordination does not increase monetary reward. This suggests that even when monetary benefits to the self are not maximized, the act of cooperating is in itself rewarding.

There is also an abundance of evidence illustrating that reciprocal actions are valued in the wake of a fairness violation. In these cases, however, violating fairness norms characteristically engenders behaviors that are construed as negative reciprocity, such as punishing the perpetrator (Fehr & Fischbacher, 2004). Nearly two decades of work demonstrates that receiving unfair offers in the Ultimatum Game is associated with increased anterior insula and anterior cingulate cortex activity, regions associated with negative emotional experiences and conflict (Chang & Sanfey, 2011; Sanfey et al., 2003; Xiang, Lohrenz, & Montague, 2013). In contrast, receiving fair offers recruits the reward network, including ventral striatum and orbitofrontal cortex (Tabibnia, Satpute, & Lieberman, 2008). These reward regions also become engaged when punishment is levied on the transgressor, suggesting that people highly value enforcing fairness norms, even when punishment comes with a monetary cost (de Quervain, Fischbacher, Treyer, & Schellhammer, 2004; Hu, Strang, & Weber, 2015; Singer et al., 2006).

Neural evidence for the value of reciprocity in altruism is less straightforward and less abundant, largely due to the fact that altruism appears to draw upon an indirect reciprocity mechanism. This tautologically requires that any expected returns from norm compliance be abstracted from the altruistic action itself (e.g., in the form of “social capital”). Accordingly, identifying the neural underpinnings of reciprocity in the domain of altruism requires observing how indirect reciprocity manifests over time, or at least between multiple individuals in an iterated task. These features make it relatively difficult to study the neural value of reciprocity within an altruistic context, and thus there is limited work on the topic. However, in the few cases in which researchers have fruitfully examined the blood-oxygen-level-dependent signal underpinning the effects of indirect reciprocity during altruistic social exchanges, evidence dovetails with work on trust, fairness, and cooperation: Altruistic decisions are influenced by indirect reciprocity motivations, which is subserved, in part, by increased caudate activity (Watanabe et al., 2014). In other words, even an indirect reciprocity mechanism that manifests across multiple individuals behaving altruistically appears to rely on regions that process reward.

In contrast, those who have broken a moral norm (oftentimes to selfishly enhance their own monetary benefit) demonstrate a different pattern of neural activity that does not reliably include reward regions. Several neuroimaging studies across multiple different social domains illustrate that the dorsolateral prefrontal cortex (dlPFC) is recruited when selfishly violating a moral norm (Baumgartner, Knoch, Hotz, Eisenegger, & Fehr, 2011; De Neys, Novitskiy, Geeraerts, Ramautar, & Wagemans, 2011; FeldmanHall, Dalgleish, et al., 2012; Ruff, Ugazio, & Fehr, 2013; Yamagishi et al., 2016). Given the role of the dlPFC in cognitive control (Greene, Nystrom, Engell, Darley, & Cohen, 2004; Mansouri, Tanaka, & Buckley, 2009; Ochsner & Gross, 2005), activation of this region during selfish decisions suggests that it may be difficult for individuals to adjudicate between options when a selfish opportunity is sufficiently tempting. Neural activity in dlPFC may therefore reflect the deployment of cognitive control to overcome concern for another’s welfare (Rilling et al., 2007). These neural data paint an emerging picture that cognitive control appears to be required to resolve self-other conflicts that ultimately favor the self.

## 4 . Emotions facilitate reciprocal behavior

Although emotion has historically been regarded as an irrational and dangerous threat to our moral calculus (Plato, 1955), the last few decades have fruitfully illustrated how emotion can play a special role in the establishment of response-dependent values and norm compliance (D’Arms & Jacobson, 1994; Phelps, Lempert, & Sokol-Hessner, 2014). Take, for instance, a situation where you contemplate cheating on your spouse. You might feel a pang of disapproval or shame upon considering such behavior. These moral emotions moderate moral standards (is it wrong if you are in an unhappy marriage?), and by extension, moral behavior (do you decide to have the affair?). In essence, the link between norm compliance and moral behavior is thought to be influenced by moral emotions (Tangney, Stuewig, & Mashek, 2007), insofar that emotional experiences can sustain one’s own compliance with moral norms and motivate enforcement of norm compliance in others (Dunning, Fetchenhauer, & Schlösser, 2012; Fehr & Gächter, 2002).

### 4.1. Self-directed emotions

Guilt and shame are emotions that are explicitly linked to promoting the interests of society rather than one’s own interests (Pizarro, 2000). These moral emotions emerge early in childhood (Vaish, 2018), and are negative evaluations of one’s own morally transgressive behavior (Eisenberg, 2000). Guilt appears to be a particularly salient motivator of reparative behavior, as it encourages people to make amends for violating moral norms, and can thus enhance how positively the transgressing person is perceived (Stearns & Parrott, 2012). Guilt-proneness consistently correlates with measures of perspective-taking and is inversely related to antisocial and criminal behavior (Tangney et al., 2007). Aligning with these findings, several neuroimaging studies have found that when describing moral transgressions, feelings of guilt are associated with neural activity in a network that corresponds with thinking about other people (Basile et al., 2011; Shin et al., 2000; Takahashi et al., 2004), which may reflect that a key function of guilt is to promote perspective-taking. In these cases, it is likely that individuals are thinking about their partner’s expectations, and thus guilt seems to exert the greatest influence on reciprocal moral actions. As guilt is associated with breaches of moral norms and social standards, the existence of guilt (or even the anticipation of guilt) is a potent motivator for upholding moral norms (Battigalli & Dufwenberg, 2007; Chang et al., 2011).

Although emotions such as guilt encourage people to avoid breaking norms, other emotions motivate people to actively comply with norms. For example, some theories propose that empathy sensitizes people to value altruism (Batson et al., 1991; Preston, 2013; Zaki, 2014). To the extent that the interplay between norms enables flexible moral action, it may therefore be the case that empathy’s primary contribution is the promotion of altruism above other norms (Rumble, Van Lange, & Parks, 2009; Zaki & Mitchell, 2011), which can be amplified by warm glow motives (Andreoni, 1990; Ashar, Andrews-Hanna, Dimidjian, & Wager, 2017; FeldmanHall et al., 2015). Indeed, recent work on extraordinary altruists demonstrates that these individuals maintain atypically high concern for the welfare of distant others (Vekaria, Brethel-Haurwitz, Cardinale, Stoycos, & Marsh, 2017), a finding that is mirrored by experimental inductions of empathy in normative populations (Klimecki, Mayer, Jusyte, Scheeff , & Schönenberg, 2016). In addition to warm glow motives, other positive emotions (such as happiness) can also actively facilitate prosocial behaviors through a reward reinforcement mechanism (Aknin, Van de Vondervoort, & Hamlin, 2018.

### 4.2. Other-directed emotions

Negative emotions such as anger and disgust arise from being treated unfairly, and are believed to motivate punishment (Pillutla & Murnighan, 1996; Srivastava, Espinoza, & Fedorikhin, 2009; Van’t Wout, Kahn, Sanfey, & Aleman, 2006). Recent work reveals that the act of punishing can alleviate the onslaught of these negative emotional experiences (Hétu, Luo, D’Ardenne, Lohrenz, & Montague, 2017). Unsurprisingly, watching people break moral norms that target other individuals can also give rise to a similar set of moral emotions, including righteous anger, indignation, contempt, and disgust (Dubreuil, 2010; Moll et al., 2002; Rozin, Lowery, Imada, & Haidt, 1999).

Contempt (the moral denunciation of others) is often expressed in response to the violation of communal codes, and is therefore a negative social evaluation of others (Tangney et al., 2007). Contempt is most often expressed by those not directly harmed by the violation, and thus deals with norm compliance from a third-party perspective. Bystanders observing an injustice can express contempt to ostracize the agent causing harm. For example, people feel contempt towards those who violate social hierarchy norms (Rozin et al., 1999). When presented with an angry, contemptuous face criticizing a norm violation, individuals report greater feelings of guilt (Giner-Sorolla & Espinosa, 2011), which can affect rates of future norm compliance. In essence, these third-party emotions are used for social policing, with the aim to minimize morally offensive behavior (Tangney, Miller, Flicker, & Barlow, 1996).

### 4.3. Atypical emotion processing

Emotion’s critical role in guiding norm compliance is even more evident when considering populations whose processing of emotions is atypical. Individuals who fail to generate an emotional arousal response before approving harmful, immoral actions illustrate how a lack of anticipatory emotional response results in behavior that is insensitive to moral norms and future consequences (Blair, 1996; Harenski, Harenski, Shane, & Kiehl, 2010; Moretto, Làdavas, Mattioli, & Di Pellegrino, 2010; Rilling et al., 2007; Shamay-Tsoory, Harari, Aharon-Peretz, & Levkovitz, 2010). For example, lesions to the medial frontal cortex typically lead to blunted emotional responding (Bechara, Damasio, & Damasio, 2000), and accumulating evidence indicates that this region is critical for evaluating emotional states and integrating this information within the context of current goal states, such as adhering to relevant social norms (Forbes & Grafman, 2010). In other words, the medial frontal cortex likely processes internal emotional signals alongside cues about social norms to help guide successful moral behavior.

Individuals diagnosed with psychopathy and conduct disorder also provide a compelling case for the intimate link between disrupted emotional responses and patterns of aberrant moral behaviors. For example, when watching others in pain, adult psychopaths, adolescents who exhibit psychopathic traits, and adolescents diagnosed with conduct disorder all show attenuated engagement of brain regions known to respond to another’s pain (Decety, Chen, Harenski, & Kiehl, 2013; Decety, Michalska, Akitsuki, & Lahey, 2009; Marsh et al., 2013). For psychopaths, these failures in appreciating the emotional aspects of a victim’s suffering has been explicitly linked to both abnormal (i.e., immoral) judgments (Young, Koenigs, Kruepke, & Newman, 2012), and an insensitivity to norms of generosity (Koenigs, Kruepke, & Newman, 2010). There is emerging research, however, that suggests these aberrant moral behaviors may also be a product of failures in processing value (Baskin-Sommers, Stuppy-Sullivan, & Buckholtz, 2016; Hosking et al., 2017; Mitchell et al., 2006). Individuals with higher levels of psychopathy cooperate less and exhibit reduced activity in orbitofrontal cortex when cooperating (Rilling et al., 2007), hinting at a causal role of reward in motivating cooperative behavior. However, given the intimate link between emotion and reward (Adolphs, 2002; Berridge & Robinson, 2003; Knutson, Adams, Fong, & Hommer, 2001; Kringelbach, 2005; Murray, 2007; O’Doherty, Kringelbach, Rolls, Hornak, & Andrews, 2001; Phelps & LeDoux, 2005), it is likely that perturbed representations of value manifest because of failures in generating an emotional response (Bechara, 2004; Bechara, Damasio, Damasio, & Anderson, 1994; Bechara, Damasio, Tranel, & Damasio, 1997), which can subsequently result in immoral behavior.

## 5 . Learning moral norms through reciprocity

Moral norms develop and are transmitted through social interactions and relationships (Ho, MacGlashan, Littman, & Cushman, 2017). The frequency with which these norms are attended and adhered to suggests that they are indoctrinated at an early age (House, 2018). Children as young as three years old can distinguish between legal and social violations (Smetana, 1983). Recent developmental research further reveals that children begin to obey norms after an authority figure illustrates they should be followed (Hardecker & Tomasello, 2017; Schmidt, Butler, Heinz, & Tomasello, 2016). Such vicarious learning appears early in the developmental trajectory, and can help facilitate the distinction between social and moral norms in young children (e.g., wearing pajamas to school versus hitting another; (Turiel, 1983). As a child’s moral calculus develops further, they begin to consider contextual factors, such as intent, provocation, and duty when evaluating which moral norms might be appropriate for the situation (Engelmann, Herrmann, & Tomasello, 2017).

Once learned, moral norms seem to be sustained through reward and punishment contingencies that are based on expectations of reciprocity (Göckeritz, Schmidt, & Tomasello, 2014; Hardecker, Schmidt, & Tomasello, 2017; Leimgruber, 2018). These expectations can be expressed both directly (e.g., monetary benefit) and indirectly (e.g., gaining social capital (Hackel & Zaki, 2018). For example, breaking certain social norms, such as wearing the wrong outfit to school, can evoke scorn and mockery from peers, and if the transgression is particularly egregious, it may even induce social rejection. Accordingly, the feedback received from others acts as a reinforcement mechanism that can dictate the adherence to (or deviance from) moral norms (Aknin et al., 2018). Over the last few years, researchers have begun to successfully apply reinforcement learning frameworks to explain how social learning unfolds. For example, prediction errors—when an actual outcome deviates from an expected outcome—allow individuals to update their expectations about the social world to align with reality (Behrens, Hunt, Woolrich, & Rushworth, 2008; FeldmanHall, Otto, & Phelps, 2018; Joiner, Piva, Turrin, & Chang, 2017; Klucharev, Hytönen, Rijpkema, Smidts, & Fernández, 2009; Montague & Lohrenz, 2007). These prediction errors, which are largely generated by the midbrain dopaminergic system and the structures it innervates (Haber & Knutson, 2010; Ruff & Fehr, 2014; Schultz, Dayan, & Montague, 1997), may drive moral learning by encoding norm violations.

In one of the first studies illustrating that norm violations generate prediction errors, researchers found that subjects in a Trust Game transferred less money to partners who violated trust (King-Casas et al., 2005). This behavior was underpinned by prediction error signals in the caudate, such that the magnitude of neural activity in response to a partner’s reciprocation (or lack thereof) tracked decisions to trust a partner with more money on the next round. Though the prediction error signal was initially observed after subjects saw feedback about whether a partner upheld a trust norm, it began to shift backward in time as subjects learned more about a partner’s trustworthiness, suggesting that subjects were developing a stable impression of their partner’s moral traits (i.e., their willingness to reciprocate). Subsequent work further decoupled monetary reward from learning about moral traits (e.g., generosity), revealing that activity in a key learning hub—the ventral striatum—indexes dissociable prediction errors when learning about money and stable moral characteristics such as generosity (Hackel, Doll, & Amodio, 2015). Moreover, prediction errors associated with learning about another’s generosity correlated with activity in a network of brain regions implicated in impression updating (including ventrolateral prefrontal cortex and right temporoparietal junction), illustrating that people find norm violations especially diagnostic in helping to form a stable impression of another’s personality (Mende-Siedlecki, Baron, & Todorov, 2013).

However, an individual’s ability to glean information about their social world (and to subsequently adaptively update their behavior) may depend on the social context and the relevant moral norm. To probe whether prediction errors are contextually modulated, researchers have dynamically manipulated moral expectations using the Ultimatum Game. When led to believe that unfair offers are ubiquitous, subjects were less willing to punish partners who break fairness norms (Sanfey, 2009), which provides compelling evidence that people adjust their behaviors according to the prevailing norms of a specific social environment. These context-sensitive decisions to punish were supported by prediction errors in canonical learning regions such as ventral striatum, substantia nigra, and VTA (Hétu et al., 2017; Xiang et al., 2013). The notion that stable impressions about another’s moral traits are dependent on moral expectations is also supported by memory research. In a study from our own lab examining how decision-making is influenced by episodic memory, we observed that people adaptively play with past partners if accurate impressions of the partner’s norm compliance have been fully encoded by rich episodic memories (Murty, FeldmanHall, Hunter, Phelps, & Davachi, 2016). Together, these results suggest that people use learned impressions of others’ moral traits to guide adaptive decision-making.

The tight coupling between norms, moral learning, and adaptive decision-making demonstrates that people use knowledge of norm violations to form impressions of others’ moral traits. Direct experience of another person’s failure to comply with norms produces prediction errors, and these errors drive fast and flexible learning about others’ moral traits, such as generosity and trustworthiness (Hackel et al., 2015; King-Casas et al., 2005). Once moral impressions stabilize, learning regions cease to track deviations from expected normative behavior (Delgado, Frank, & Phelps, 2005). As norm violations provide diagnostic information about others’ traits (Mende-Siedlecki et al., 2013), stable impressions can guide optimal choices by enabling people to affiliate with those who are likely to be rewarding social partners and to avoid those who are likely to be unrewarding (Murty et al., 2016). In fact, these moral impressions can weigh so heavily on social decisions that people choose not to cooperate with a stranger if they know that the stranger is friends with a norm violator (Martinez, Mack, Gelman, & Preston, 2016).

## 6. Integration with other theories

While we posit that moral decision-making is largely motivated by four fundamental norms, other prominent theories have argued that a number of additional norms are critical for successful socialization (Moral Foundations Theory; Haidt, 2007), or that all moral behaviors can be reduced to a single motivation—the desire to reduce harm (Theory of Dyadic Morality; Schein & Gray, 2017). Here, we have tried to strike a balance between parsimony and explanatory power. For example, Moral Foundations Theory may place undue weight on certain norms (e.g., purity) that are less represented in many everyday moral quandaries. On the other hand, the Theory of Dyadic Morality is parsimonious by its very nature. Although we would agree that many moral situations can be perceived through a lens of harm, such an account can be overly restrictive when trying to explain the wide range of findings in psychology, economics, and neuroscience.

By allowing the findings from psychology and neuroeconomics to guide us, we have highlighted reciprocity as a common mechanism that motivates adherence to a discrete suite of moral norms. The idea that reciprocity provides a unifying principle for social behavior is not new (Berg et al., 1995; Bolton & Ockenfels, 2000; de Waal & Luttrell, 1988; Fehr & Gächter, 2000b; Gouldner, 1960). Early models such as Social Exchange Theory suggested that reciprocity is a universally held principle (Gouldner, 1960), and that high-quality relationships can emerge and flourish through reciprocal actions (Cropanzano & Mitchell, 2005; Thibaut & Kelley, 1959). We build on this work, examining how this one simple mechanism can explain people’s adherence to a set of specific moral norms, and how these moral norms collectively provide an overarching framework for understanding moral behavior across a variety of domains.

## 7. Conclusions

Moral norms facilitate harmonious interpersonal exchanges by providing people with a set of common expectations. Here we highlight four norms—fairness, altruism, trust, and cooperation—that we believe to be the most foundational for successful social living. By discussing the ways in which these norms can shape behavior, we offer an account for the proximate psychological mechanisms motivating moral norm compliance: Reciprocity. People comply with moral norms because they have the direct or indirect expectation that others will also adhere to these norms, and because they believe that norm violations may have negative repercussions for the future well-being of both specific individuals and entire societies. Activation in the brain’s reward network supports active adherence to these moral norms, suggesting that people find value in complying with norms and engaging in reciprocal behaviors with others. In addition, we examine how aversive moral emotions such as contempt and guilt facilitate norm enforcement by devaluing selfish, norm-violating actions. Finally, we review evidence that learning about norm violators depends on a network of brain regions that encode for reward and violated expectations of receiving reward, suggesting that people learn about others’ social value through a reinforcement learning mechanism.

The degree to which humans act fairly, help, trust, and cooperate is often viewed as a puzzle across an array of disciplines. Some of the deepest thinkers in human history, including Adam Smith, Jean-Jacques Rousseau, and Charles Darwin, have attempted to provide accounts of how social norms dictate appropriate behaviors in nearly every aspect of human life, from the trivial (e.g. wearing the correct attire to a wedding) to the deeply consequential (e.g. punishing a criminal with the death sentence). However, few accounts have successfully reconciled two seemingly contradictory features of norm compliance: Although social norms are pervasive and often perceived as inflexible in nature, the degree to which an individual adheres to these norms produces malleable and context-specific behaviors. Emerging research in moral psychology and neuroscience elucidates how norms are supported by the simple cognitive mechanism of reciprocity. Reciprocal behavior is stable enough to support interpersonal exchanges between strangers, yet flexible enough to accommodate adaptive behavior across a range of social environments. We provide a unifying framework for understanding how a wide variety of putatively unrelated moral behavior—helping a homeless person, getting angry at a fraudster, asking a stranger at the library to look after your computer while you take a call—are supported by expectations of reciprocity and the associated neural encoding of reward.

## Conflicts of Interest:

None.
